# Pemphigus vegetans developing after Mohs micrographic surgery and cryotherapy^☆^^☆☆^

**DOI:** 10.1016/j.abd.2020.07.018

**Published:** 2021-05-15

**Authors:** Nathan Jetter, Felipe Bochnia Cerci, Karan Pandher, Aleksandar L. Krunic

**Affiliations:** a Department of Dermatology, University of Illinois College of Medicine, Chicago, USA; b Department of Dermatology, Hospital de Clínicas, Universidade Federal do Paraná, Curitiba, PR, Brazil; c Chicago Medical School, Rosalind Franklin University, Chicago, USA; d Department of Dermatology, School of Medicine, Northwestern University Feinberg, Chicago, USA

Dear Editor,

Pemphigus vegetans (Pveg) is a subtype of pemphigus vulgaris (PV), characterized by flaccid blisters which become erosions and vegetating plaques, typically in the intertriginous areas, face, and scalp.^1^ Histologically it presents as pseudoepitheliomatous hyperplasia (PEH) associated with suprabasal acantholysis.

Trauma-induced pemphigus is rare following surgical procedures, with only a few reports occurring after Mohs micrographic surgery (MMS).^2^, ^3^, ^4^, ^5^ Herein, a patient who developed Pveg after MMS for squamous cell carcinoma (SCC) of his chest and after cryosurgery for actinic keratosis (AKs) at his temple and forehead is presented. To the authors’ knowledge, this is the first report of Pveg arising within either an MMS site or site treated by cryosurgery.

An 81-year-old caucasian male presented with several months of an erythematous hyperkeratotic nodule on the right anterior chest. Biopsy confirmed well-differentiated SCC and the patient underwent MMS for tumor removal. Concomitantly he was submitted to cryosurgery on his left temple and forehead for AKs. The postoperative course, initially unremarkable, was complicated by poor wound healing, oozing and discharge from the wound sites, as well as maceration at the periphery. Wound cultures grew *Pseudomonas aeruginosa*, but the patient failed to respond to systemic and topical antibiotics. Two months after MMS, the patient was noted to have erosions and hyperkeratotic vegetating plaques expanding circumferentially from the procedure sites (Fig. 1). Biopsy from the chest demonstrated PEH and suprabasal acantholysis suggesting Pveg. Direct immunofluorescence showed deposits of IgG and C3 in the intercellular spaces of the epidermis, compatible with pemphigus (Fig. 2). Indirect immunofluorescence revealed autoantibodies against the epidermal cell surface at a titer of 1:40. ELISA showed anti-desmoglein (DSG) 1 antibody titer at 131.4 and anti-DSG3 antibody titer at 34.2 (>20 is positive for both).

**Figure 1:**
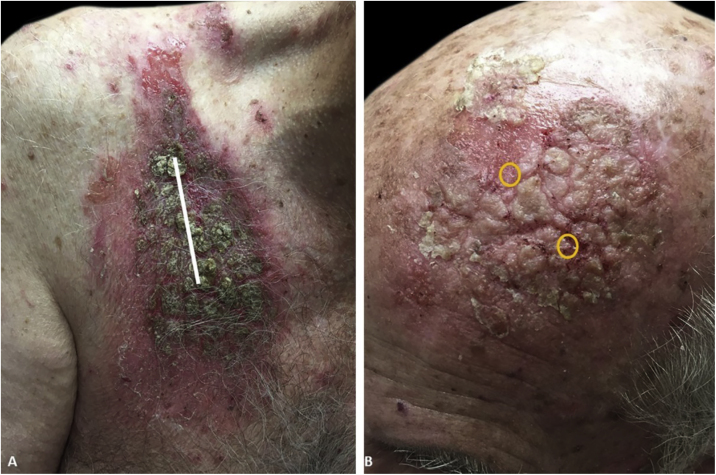
(A), Vegetative, hyperkeratotic, eroded plaques on the right chest (Mohs surgery site). The site of primary closure has been superimposed with a white line. (B), Left temple/forehead (cryosurgery site). Sites of original trauma have been superimposed with orange circles.

**Figure 2:**
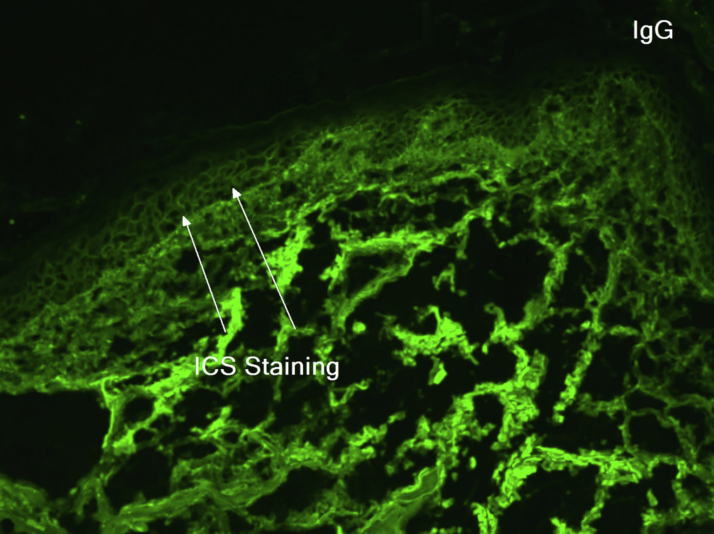
Direct immunofluorescence on skin biospy demonstrating IgG fluorescence around keratinocytes (white arrows).

The immunofluorescence and histopathology results along with the clinical presentation were consistent with Pveg. The patient was started on oral dexamethasone 0.15 mg/kg and azathioprine 150 mg daily and topical steroids, with complete resolution of the skin lesions in the due course (Fig. 3).

**Figure 3:**
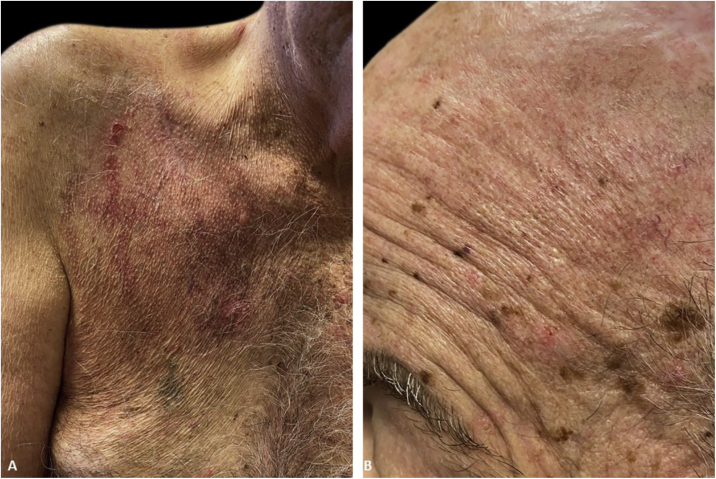
Complete healing 3 weeks after initiation of immunosuppressive therapy with residual erythema. A linear scar in the middle of the right chest can now be seen in the center.

Trauma-induced PV has been described after major general surgical procedures (abdominal, chest, orthopedic) and dental procedures.^1^ Out of 36 cases of surgically induced-PV, thirteen were in patients without pre-existing pemphigus. The literature review revealed only 2 cases of PV and 2 cases of PF occurring after MMS, and one case of PF after cryosurgery for AKs.^2^, ^3^, ^4^, ^5^ In most cases of pemphigus following MMS, including this present case, the patients presented with unremarkable healing in the immediate postoperative period followed by the development of erosions, scaling, oozing, and desquamation after 4–5 weeks post-procedure simulating wound infection or contact dermatitis.^2^, ^4^, ^5^ All cases required a high index of suspicion with biopsy confirmation and immunofluorescence testing.

Several mechanisms have been proposed to explain the induction of pemphigus and Koebnerization of pre-existing pemphigus by surgical trauma, and the process is likely to be multifactorial.^1^ Epidermal injury may expose DSG 1 and 3 and lead to new autoantibody formation in genetically susceptible patients or to activation of pre-existing antibodies already present in low (subclinical) titers.^1^, ^2^, ^4^ Furthermore, surgical trauma may link antigens not related to pemphigus but capable of immune response to pemphigus antigens through the process of epitope spreading.^1^, ^3^ These factors could potentially explain the long latency period (15 weeks) for pemphigus induction in non-dermatology surgery procedures where there is much less injury to the epidermis. With MMS, cryosurgery or shave biopsies there is more damage to the skin layers and at the dermo-epidermal junction, producing higher concentrations of released antigens (DSG 1 and 3) leading to more efficient epitope spreading and faster and stronger immunological response.^1^, ^3^ Finally, SCC itself could develop an expression of DSG 1 and 3 and trigger an autoimmune response.

Pveg poses a further diagnostic challenge as it has histological similarities with SCC due to the presence of PEH. Besides being associated with suprabasal acantholysis, PEH in Pveg cases is of adnexal (follicular) origin, confined to the epidermis and dermis, with minimal atypia, rare mitoses, and absent individual keratinocyte necrosis.

In patients who have known bullous disease reconstruction of MMS wounds should be simple, and 2^nd^ intention or partial closure should be considered.^4^ Some authors recommend increasing oral immunosuppression in the immediate postoperative period.^5^

In patients like ours who present without previous history of bullous disease, Pveg associated to the Mohs surgery and the criotherapy must have a high suspicion on the appearance of non-healing wounds or localized inflammation occurring several weeks after the procedure.

## Financial support

None declared.

## Authors’ contributions

Nathan Jetter: Participation in the conception and planning of the study; obtaining, analyzing, and interpreting the data; writing; approval of its final version.

Felipe Bochnia Cerci: Analyzing and interpreting the data; writing; approval of its final version.

Karan Pandher: Participation in the conception and planning of the study; obtaining, analyzing, and interpreting the data; writing; approval of its final version.

Aleksandar L. Krunic: Participation in the conception and planning of the study; obtaining analyzing and interpreting the data; critical review of the manuscript; approval of its final version.

## Conflicts of interest

None declared.
